# Summer Noises

**Published:** 1901-05

**Authors:** 


### SUMMER NOISES.

WITH the approach of summer with its open doors and windows, the inhabitants of the large cities will be annoyed and disturbed by the multitudinous noises incident to city life. While many of these cannot be avoided, yet many can be curtailed and the most flagrant certainly held in check. The JOURNAL has repeatedly called attention to the unnecessary noises, and now at the beginning of the Pan-American summer when a horde of visitors and strangers is expected in the city, street noises will be necessarily augmented. Late at night and in the early morning are the times of day when the loud street noises especially jar on one's sensitive nature.

These are the hours when many of our visitors are returning from the exposition grounds, and although the great majority of them will be quiet and decorous, yet some among them will insist on making noisy demonstrations unless otherwise instructed. All of us cannot jubilate at the same time. While some carouse others must work, and those who do work must have freedom from annoyance during certain parts of the day.

Perfect health demands a certain amount of quiet and repose not alone at night during sleeping hours, but during waking hours as well. The active working brain is just as much in need of rest as are the muscles of the different portion of the body, and, perhaps, the most potent factor in accomplishing this need is to secure freedom from noise. The brain, as a whole, cannot be in perfect repose when certain centers are in a state of action or irritation. The association fibers, connecting all the various centers and parts of the brain, convey the irritation from one center to another and soon the whole brain is in sympathy with the disturbed and agitated region.

The dangers arising from noises are two-fold. First, at the tympanum, where a certain amount of hyperemia and hypertrophy are liable to result, sometimes even rupture of the tympanum, producing partial or total deafness, and secondly, at the auditory center, where fatigue is apt to follow the incessant activity and irritation of these centers, and a general brain tire or brain exhaustion is liable to result.

